# Clinical improvement following an integrative iboga microdosing protocol in post-concussive and hypoxic brain injury syndromes: a case series

**DOI:** 10.3389/fphar.2026.1840956

**Published:** 2026-06-03

**Authors:** Burton J. Tabaac, Robin L. Carhart-Harris, Teresa Yung

**Affiliations:** 1 Department of Neurology, University of Nevada Reno School of Medicine, Reno, NV, United States; 2 Department of Neurology, Carson Tahoe Health, Carson City, NV, United States; 3 Departments of Neurology and Psychiatry, University of California San Francisco, San Francisco, CA, United States; 4 Department of Psychology, Meridian University, Petaluma, CA, United States

**Keywords:** accelerated experiential dynamic psychotherapy (AEDP), iboga microdosing, neurological rehabilitation, neuroplasticity, post-concussive syndrome, psychedelic-assisted therapy, traumatic brain injury, hypoxic brain injury

## Abstract

**Background:**

Traumatic brain injury (TBI) can result in prolonged post-concussive syndrome and chronic hypoxic-ischemic brain injury (HIBI) sequelae remains therapeutically challenging with the persistence of significant neurological and cognitive impairments. While conventional treatments often provide limited relief, emerging research explores alternative therapeutic interventions, including psychedelic compounds combined with therapeutic interventions.

**Objectives:**

This naturalistic case series examines clinical observations following an integrative, participant-directed iboga-containing microdosing protocol paired with Accelerated Experiential Dynamic Psychotherapy (AEDP) in three individuals with persistent neurologic symptoms after traumatic brain injury (TBI) or hypoxic-ischemic brain injury.

**Methods:**

Three participants completed a 6 week protocol using Tabernanthe iboga root bark biomass (participant-directed titration 0.1–1.0 g/day, 4 days-on/3 days-off). Quantitative qNMR/HPLC analysis (University of Cape Town) demonstrated approximately 3.845% ibogaine content by mass, yielding estimated ibogaine-equivalent exposure of 3.8–38.5 mg/day. All administration utilized whole root bark biomass only. Weekly AEDP psychotherapy and supportive nutraceuticals were provided concurrently.

**Patient one:**

A 43 year-old man with TBI sustained in a motorcycle accident. Patient Two: A 40-year-old woman with chronic hypoxic brain injury sustained during an avalanche burial event. Patient Three: A 19 year old woman with TBI sustained in a motor vehicle accident.

**Results:**

All three patients demonstrated progressive neurological recovery over the 6-week microdosing iboga protocol with two of the patients declaring complete symptom remission at a long term follow up assessment. Initial reported symptoms included a constellation of daily headaches, episodic migraines, disequilibrium, irritability, mood swings, fatigue, brain fog, sleep disruptions, and loss of interest in typical life activities. At the conclusion of the protocol, and at long term follow-up visits, patients felt able to discontinue all prescription medications for symptomatic treatment, reporting absence of severe migraine headaches, resolution of brain fog, fatigue, irritability, and stabilized mood, with the ability to return all regular activities with a renewed enthusiasm for life. All patients provided consent to share their significant clinical and therapeutic improvement journey in this publication.

**Safety considerations:**

The microdosing protocol was carefully implemented with rigorous screening to mitigate potential cardiac and neurological risks associated with iboga administration, including medical background screening for potential drug interactions, and past medical history contraindications including heart conditions and/or the concomitant administration of selective serotonin reuptake inhibitors (SSRIs).

**Conclusion:**

This naturalistic case series provides hypothesis-generating observations regarding possible clinical improvement following an integrative iboga-containing intervention paired with psychotherapeutic and supportive care. The findings do not establish causality or iboga-specific efficacy and should be interpreted within the context of multimodal therapeutic exposure and substantial methodological limitations. These preliminary observations suggest that an integrative iboga microdosing protocol, in association with psychotherapeutic and supportive care, may be linked to meaningful improvements in prolonged post-concussive symptoms. As a hypothesis-generating case series, these findings warrant further investigation in controlled trials to establish causality and specificity.

## Background

Traumatic brain injury (TBI) encompasses a spectrum of severity, with mild TBI (mTBI), commonly referred to as concussion, representing the majority of cases. Acute concussion symptoms typically resolve within 7–14 days in most individuals, though a subset experience lingering effects. Prolonged or persistent post-concussive symptoms (also termed persisting symptoms after concussion or previously post-concussion syndrome [PCS]) are defined as a constellation of physical (e.g., headache, dizziness, fatigue), cognitive (e.g., concentration difficulties, memory issues), emotional (e.g., irritability, anxiety), and sleep-related complaints that continue beyond the expected recovery period. Consensus guidelines and clinical sources commonly consider symptoms persisting beyond 3 months as persistent PCS, though some frameworks (e.g., recent international consensus statements) describe persisting symptoms as those failing to improve or worsening after 4 weeks, with 10%–30% of concussion cases affected. In this case series, we focus on individuals with prolonged post-concussive symptoms persisting for months to years following mTBI who presented with significant functional impairment despite standard care.

In recent years, there has been a burgeoning interest in the potential therapeutic applications of psychedelics, with researchers exploring their effects on cognition, emotion, and wellbeing (de Vos et al., 2021; Carhart-Harris and Friston, 2019; Carhart-Harris, 2018). Among these substances, ibogaine, a psychoactive compound derived from the roots of the West African shrub *Tabernanthe iboga*, has emerged as a subject of intense investigation (Brown and Alper, 2018; Noller et al., 2018). Initially used by the African Forest people, then passed to the Bwiti, a spiritual tradition and cultural group primarily in Central Africa, iboga is traditionally used in spiritual practices. Ibogaine has captured attention for its potential to induce altered states of consciousness and therapeutic effects, particularly in addiction treatment (Mosca et al., 2023; Loizaga-Velder and Loizaga Pazzi, 2024).

Previously published research has revealed promising results in mitigating substance dependence, with studies demonstrating ibogaine’s potential to facilitate detoxification and support abstinence (Luz and Mash, 2021; Mash et al., 2018). The compound’s complex pharmacokinetics and multifaceted neurological interactions have intrigued researchers, particularly its effects on dopaminergic circuits and neuroplasticity (Maciulaitis et al., 2008; Ma et al., 2023; Cameron et al., 2021). For instance, studies have shown that ibogaine administration can modulate growth factors like GDNF and BDNF in brain regions involved in mesocorticolimbic and nigral dopaminergic circuits (He and Ron, 2006).

However, the exploration of the use of iboga in its full form and sub-perceptual doses in a microdosing context remains uncharted territory in the western medical field, necessitating a nuanced understanding of its effects on various psychological dimensions (Kuypers, 2020). Psychedelic substances have long been recognized for their capacity to elicit profound alterations in perception, mood, and cognition (de Vos et al., 2021; Kuypers, 2020). Understanding these effects is crucial not only for unraveling the intricacies of the human mind but also for tapping into the neurological and therapeutic potential of these compounds.

Ingestion of iboga comes from South African Pygmy and Bwiti traditional healing practices, where iboga has been used for thousands of years for its healing properties. There it is understood not merely as a chemical compound but as a plant medicine, also known as the “holy wood” that facilitates healing on physical, psychological, and spiritual levels. The dosing protocol was shared and informed by the expertise of two South African Sangomas—traditional healers with over a decade of experience working with iboga. These practitioners have honed a culturally rooted and empirically guided approach to microdosing with iboga, emphasizing its integration within holistic healing frameworks.

Traditional ethnomedicine perspectives, including those of the practitioners who created the original protocols, regard iboga not simply as a compound to be extracted and standardized, but as an intelligent plant medicine with inherent wisdom in facilitating a holistic approach to healing. While maintaining scientific objectivity, acknowledging this traditional framework may offer insights into optimal therapeutic applications.

The addition of therapeutic guidance and supervision was added to these cases. This collaboration highlights the value of traditional knowledge, modern day therapeutic practices, and western scientific clinical practice, bridging indigenous wisdom, psychological and therapeutic practice with contemporary neuroscience. The integration of traditional wisdom with modern medical oversight represents an emerging model of complementary care that honors both empirical and traditional knowledge systems. The patients adhered to a dosing schedule of 4 days on and 3 days off, allowing for both potential benefits and mitigation of adverse effects associated with prolonged use (Winkelman, 2014; Mash et al., 1998). This approach seeks to explore the subtler, long-term effects of iboga microdosing while minimizing the risk of overwhelming perceptual changes.

It is important to note the potential risks associated with iboga. Previous studies have documented concerns about cardiac effects, including QT interval prolongation and potential arrhythmias (Koenig and Hilber, 2015; Litjens and Brunt, 2016; Knuijver et al., 2024; Kubiliene et al., 2008; Knuijver et al., 2022; Mash et al., 2000). Additionally, neurological considerations such as potential Purkinje cell degeneration have been observed in some research (O'Hearn and Molliver, 1993; Vocci and London, 1997). Therefore, careful screening and monitoring are critical in any iboga-related intervention (Winkelman, 2014; Mash et al., 1998).

The current case series focuses on a unique application of iboga microdosing in the context of traumatic brain injury (TBI) rehabilitation, building upon emerging research exploring psychedelic interventions for neurological recovery, particularly in veteran populations (Brody and Siddiqi, 2024; Cherian K. N. et al., 2024; Khan et al., 2022; Plummer et al., 2025).

## Methods

### Screening

The participants completed a pre-screen assessment including health, mental health, and substance use. Health questions include heart condition, physical activity, cerebellar dysfunction, epilepsy, brain disease, dementia, impaired kidney or liver function. Mental health questions include acknowledgement of schizophrenia, bipolar disorder, depersonalization and/or derealization disorder, and psychosis or any acute confusional state. Substance use questions include use of any psychiatric drugs (anti-anxiety medication, antidepressants, and mood stabilizers), use of opiate drugs (heroin, opium, morphine, oxycontin, suboxone, methadone), use of benzodiazepines (Xanax, Valium, Ativan), and use of any drugs for sleeping or staying awake. All data collected in assessments are collected electronically and secured in a secure, password-protected database.

### Participants and recruitment

Participants were self-referred or referred by clinicians, community networks, or peer recommendations, seeking novel integrative options for persistent post-concussive symptoms that had not adequately responded to standard medical and rehabilitative care. Recruitment was passive and occurred through word-of-mouth, and professional referrals. Inclusion criteria included: (1) documented history of traumatic brain injury (mild to moderate severity), (2) prolonged post-concussive symptoms or post-hypoxic brain injury symptoms persisting for at least 3 months (and typically much longer) despite conventional treatments, (3) age 18 years or older, (4) capacity to provide informed consent, and (5) medical stability for outpatient integrative care. Exclusion criteria included: active psychosis or severe untreated psychiatric illness, current substance dependence (other than nicotine), contraindicating medications (e.g., QT-prolonging drugs), severe cardiac disease, pregnancy, or inability to engage in psychotherapy. All participants paid for the therapy component portion of the intervention on a sliding-scale basis, ranging from USD $1,100 to $1,500, which covered integrated psychotherapy sessions (weekly counseling, parts work, AEDP, and integration). No financial incentives or subsidies were provided, and fees were structured to ensure accessibility while supporting the therapeutic practice. Screening was comprehensive and conducted by the treating clinician (the corresponding author, a board-certified physician with expertise in psychedelic-assisted therapies), in consultation with participants’ existing medical providers as needed. The process included: a detailed medical and psychiatric history review; specific cardiac risk assessment (beyond a general ‘heart condition’ question) encompassing personal/family history of arrhythmias, QT prolongation, syncope, or sudden cardiac death; review of current medications for potential interactions; baseline vital signs measurement; and, where clinically indicated, 12-lead ECG to evaluate QTc interval and other abnormalities. Participants with any concerning cardiac findings were referred for cardiology consultation prior to proceeding. This rigorous screening ensured participant safety given known cardiovascular risks associated with ibogaine-containing preparations. The iboga was obtained by the patients directly from the traditional healers.

The therapy portion intervention was administered in the United States, in the state of Nevada. Participants were recruited from the United States. No non-US participants required translators, as all sessions were conducted in English with native fluent speakers.

Ethics and Informed Consent: This case series describes naturalistic observations from individuals who independently elected to engage in an iboga-containing protocol outside formal clinical care. Data were collected and de-identified for scholarly analysis. As no intervention was assigned by investigators and no prospective human-subject experimentation occurred, formal IRB review was not required under institutional policy. All participants provided written informed consent for publication of de-identified clinical information.

To ensure the highest ethical standards, the work was conducted in full accordance with the ethical principles outlined in the Declaration of Helsinki (2013 revision), with particular emphasis on: 1. Respect for persons (autonomy through voluntary participation and informed consent processes), 2. Beneficence (maximizing potential benefits while minimizing risks via careful screening and monitoring), and 3. Justice (equitable access to the integrative protocol without coercion). All participant data were de-identified prior to analysis and reporting, with stringent measures to protect privacy and confidentiality in line with HIPAA Safe Harbor guidelines.

Participants provided written clinical informed consent for the integrative iboga microdosing and psychotherapeutic intervention as part of standard care in a therapeutic setting. Subsequently, for inclusion in this observational case series, separate research informed consent was obtained, allowing use of anonymized clinical data for publication. The research consent process included full disclosure of study aims, risks/benefits, confidentiality measures, and voluntary withdrawal rights.

### Intervention

The iboga preparation used in this protocol was a natural root bark product derived from *Tabernanthe iboga*, containing ibogaine and other naturally occurring alkaloids (not a synthetic ibogaine hydrochloride preparation). The material was sourced directly from a Pygmy family in the Congo who cultivate and traditionally harvest the plant in accordance with sustainable and culturally respectful practices. This sourcing approach prioritizes ethical, community-based supply chains in the plant’s region of origin, minimizing risks associated with unregulated or adulterated products commonly encountered in global markets. The supplier provided a Certificate of Analysis (COA) documenting third-party laboratory testing for alkaloid content (including quantified ibogaine percentage), purity, and absence of contaminants such as heavy metals, pesticides, microbial pathogens, and adulterants. The COA was reviewed by the treating clinician prior to use to confirm suitability for therapeutic application. Participants received the product in measured, standardized doses as part of the microdosing protocol, with all administration occurring under direct clinical supervision or guided self-administration with follow-up monitoring.

Participants obtained approximately 20 g of Tabernanthe iboga root bark biomass for the 6 week protocol directly from the Sangoma healers. Dosing followed an individualized participant-directed titration framework utilizing approximately 0.1–1.0 g/day on a 4 days-on, 3 days-off schedule. Third-party qNMR and HPLC Certificate of Analysis testing demonstrated approximately 3.845% ibogaine content by mass within the root bark biomass. Participants utilized biomass/root bark only and did not utilize purified ibogaine hydrochloride or concentrated extracts. Participant characteristics, injury details, iboga dosing parameters, and overall clinical course are summarized in Table 1.

**TABLE 1:** Structured clinical summary.

Patient	Classification	Symptom duration	Iboga dose range	Follow-up	Interpretation
1	Persistent PCS (TBI)	11 months	0.1–1.0 g/day	Jan 2025	Substantial improvement (multimodal)
2	Chronic HIBI	7 years	0.1–1.0 g/day	Jan 2025	Complete symptom resolution
3	Persistent PCS (TBI)	3 months	0.1–1.0 g/day	June 2025	Partial remission with relapse

### Monitoring and support

The weekly counseling component addressed the psychological dimensions of recovery and enhanced the therapeutic efficacy of the intervention. Weekly sessions were held via virtual connection to the therapist, while monitoring adherence to the microdosing regimen. This was conducted for the duration of the 6 week treatment paradigm. This case was supervised in accordance with direction from the Sangoma healers.

Literature Review: The narrative synthesis of existing literature incorporated in this manuscript was informed by searches of major databases including PubMed/MEDLINE, Google Scholar, PsycINFO, and Scopus, conducted through December 2025. Search terms included combinations of ‘ibogaine’, ‘iboga’, ‘psychedelic’, ‘traumatic brain injury’, ‘TBI’, ‘post-concussive syndrome’, ‘persistent post-concussion symptoms’, ‘concussion’, ‘neuroplasticity’, ‘GDNF’, ‘BDNF’, and related terms. Inclusion focused on peer-reviewed articles, reviews, and case reports relevant to psychedelic (particularly ibogaine/iboga) mechanisms or applications in TBI, post-concussive symptoms, neuroprotection, or related neuropsychiatric conditions. Exclusion criteria eliminated non-English publications, non-peer-reviewed sources, and studies unrelated to brain injury or psychedelics. Key studies were selected based on relevance to the clinical observations in our case series, with emphasis on high-impact preclinical mechanistic work and recent observational human data (e.g., ibogaine in veterans with TBI).

## Case report: patient one

### Patient information and history

A 43 year-old man was referred to the neurology clinic in April 2024 following a traumatic brain injury sustained during a motorcycle accident in February 2024. The patient experienced loss of consciousness at the time of impact, sustained helmet damage, and reported a 24 h period of anterograde amnesia. The patient’s initial neuroimaging occurred approximately 1-month post-injury.

### Clinical findings

#### Initial presentation (April 2024)

Upon initial evaluation, the patient presented with a complex post-concussive syndrome characterized by daily headaches with at least 1 day per week of continuous cephalic pain. The patient described a dull occipital headache pattern with intermittent throbbing. Associated symptoms included nausea, photophobia, right-sided ocular pressure without visual disturbance, intermittent disequilibrium, lightheadedness, and diffuse fatigue more pronounced in the upper extremities. The patient’s spouse reported noticeable mood lability and affective changes.

Sleep patterns were disrupted, with the patient averaging approximately 6 h of sleep nightly with maintenance insomnia. Neurological examination revealed no focal sensory deficits. Initial management included supplementation with N-acetylcysteine (NAC) and Coenzyme Q10, and a prescription for amitriptyline (Elavil) 10 mg nightly. Additional interventions included Aimovig injections monthly, magnesium L-threonate for insomnia management, and recommendations for minimal screen time, proper hydration, an anti-inflammatory diet, and avoidance of smoking and alcohol.

From a psychological perspective, the patient reported significant emotional challenges, including grief over loss of identity, severely impacted physical capability, major lifestyle shifts, and diminished access to meaningful activities. The patient experienced multiple daily anger outbursts and depression stemming from inactivity and stressed family dynamics.

### Diagnostic assessment

An MRI brain with and without contrast performed in March 2024 revealed scattered small foci of hyperintense FLAIR/T2 signal predominantly affecting the deep white matter of both frontal lobes. A subtle area of confluent signal was observed in the juxtacortical white matter of the precentral gyrus near the vertex. These imaging features were consistent with nonspecific gliosis. Notably, there was no hemosiderin deposition detected, no encephalomalacia along the polar regions of cerebral hemispheres, and no evidence of diffuse axonal injury.

### Clinical course and intervention

#### First follow-up (July 2024)

At a 2 month neurology clinic follow-up visit the patient showed minimal symptomatic improvement, with heat-provoked severe headaches impacting work functionality. The patient discontinued amitriptyline due to adverse effects of irritability and excessive drowsiness and did not appreciate any benefit from magnesium L-threonate administration. Treatment was modified to include sensory deprivation tank therapy and Lion’s Mane supplementation (500 mg daily), while continuing Aimovig injections monthly.

#### Iboga microdosing protocol initiation (July 2024)

Following limited improvement with conventional interventions, the patient was introduced to an iboga microdosing protocol beginning July 2024, with weekly therapeutic sessions performed virtually, scheduled for the subsequent 6 weeks.

#### Therapeutic progression

The therapeutic intervention combined iboga microdosing with Accelerated Experiential Dynamic Psychotherapy (AEDP), a somatic-focused approach. Weekly sessions were conducted as follows:

##### Session 1 (July 2024)

Established therapeutic rapport and introduced the iboga protocol, including ritual and spiritual connection aspects. Therapeutic intervention focused on holistic assessment of the patient’s life history, interpersonal relationships, emotional management, and processing of grief related to identity changes following TBI. The patient was instructed to establish a ritualized morning routine incorporating intention setting, gratitude practice, visualization, mindfulness, and connection with iboga.

##### Session 2 (July 2024)

The patient reported a shift in headache location from occipital to frontal regions with reduced intensity during daytime hours. Therapeutic work explored the patient’s historical difficulties with emotional expression, feelings of unworthiness, and family dynamics that affected trust development since childhood. The therapist provided guidance on observing thought patterns and behaviors that might be amplified through the iboga protocol.

##### Session 3 (August 2024)

The patient detailed explosive anger episodes and their impact on family dynamics. Exploration of childhood experiences revealed adverse early experiences including physical punishment, anxiety manifesting as perceptual distortions, i.e., “walls closing in”, and emotional isolation. The therapeutic approach incorporated somatic therapy, focusing on physical sensations in the stomach and chest associated with sadness and confusion.

##### Session 4 (August 2024)

The patient reported significant improvement, noting headaches were now limited to evening hours or occasionally during sleep, with decreased intensity. Physical improvements included reduced muscle aches, decreased fatigue, and increased energy. The patient reported engaging in physical activities, e.g., hiking, and emotionally connecting activities with his son. Family relationships improved from a “grin and bear it” attitude to experiences of enjoyment and enthusiasm. The patient acknowledged improved interoceptive awareness regarding stress and anger, with enhanced self-regulation through breath work and somatic awareness.

##### Session 5 (August 2024)

Continued improvement was noted with fewer headaches, decreased fatigue, and sustained energy throughout the day. The patient reported expanding his activity level, including hiking, and developing insight into transgenerational patterns of emotional behavior, particularly regarding his son. Morning mindfulness practices continued with reported deeper connection to “something greater,” earlier waking, increased readiness for daily activities, and deeper feelings of peace. The patient acknowledged greater understanding of the connection between childhood trauma, stress, and physical pain manifestations. AEDP therapy focused on accessing somatic affect, processing childhood pain related to parental relationships, and expanding emotional tolerance through imaginal portrayal techniques.

##### Session 6 (September 2024)

The patient reported occasional musculoskeletal discomfort in the right lower back and shoulder. Anger outbursts had completely resolved, compared to multiple daily episodes prior to treatment. Headaches had decreased to approximately one every 2 weeks, compared to daily occurrences pre-intervention. The patient reported acceptance of lifestyle changes and increased enjoyment in new activities with his son. The final session focused on maintaining mindfulness and somatic awareness practices.

### Follow-up assessment (August 2024)

After initiation of the iboga microdosing protocol, the neurological clinic follow-up visit in mid-August confirmed gradual recovery with improved muscle strength and energy levels. Headaches persisted albeit with notably reduced intensity and frequency. The patient continued Aimovig injections for headache prevention alongside complementary alternative interventions aforementioned.

### Final clinical follow-up (November 2024)

At a 9-month neurology clinic post-injury follow-up, the patient reported profound and significant clinical improvement with substantially reduced muscle fatigue and headaches characterizing symptoms as “very rare and very subtle.” The patient self-discontinued all prescribed medications by this time.

### Extended follow-up (January 2025)

At an 11 month post-injury follow up assessment, status post 5 months since completion of the microdosing iboga protocol, the patient reported mild headaches occurring only once every couple of months, with occasional mild fogginess in the occipital region occurring a few times per week. Sleep had normalized with resolution of maintenance insomnia. The patient described his emotional state as “better than ever, and better than before the accident,” with improved resilience and emotional regulation. Physical symptoms had largely resolved with absence of muscle fatigue, increased motivation, and excitement about returning to pre-injury activities.

### Clinical outcome

The patient’s recovery trajectory demonstrated significant resolution of post-concussive symptoms over an 11-month period following a traumatic brain injury (TBI) sustained in a motorcycle accident. The integration of conventional neurological care with a 6 week iboga microdosing protocol and Accelerated Experiential Dynamic Psychotherapy (AEDP) therapy effectively addressed both the neurophysiological and psychological sequelae of TBI. Notable improvements were observed across multiple domains, with the patient reporting a quality of life surpassing his pre-injury baseline.

1. Physical Symptoms: The patient experienced near-complete resolution of physical symptoms that initially dominated his clinical presentation. Daily headaches, characterized by dull occipital pain with intermittent throbbing, nausea, photophobia, and right-sided ocular pressure, decreased significantly in frequency and intensity. By the November 2024 follow-up, headaches were described as “very rare and very subtle,” and by January 2025, they occurred only once every couple of months with mild occipital fogginess a few times weekly. Muscle fatigue, particularly pronounced in the upper extremities, and disequilibrium resolved completely, enabling the patient to resume physical activities such as hiking. Sleep patterns normalized, with maintenance insomnia resolving, allowing consistent 7–8 h of restful sleep nightly compared to the initial 6 h with frequent disruptions. Energy levels increased substantially, supporting a return to an active lifestyle without the diffuse fatigue that previously limited daily functioning.
2. Cognitive Symptoms: Cognitive impairments, including brain fog and confusion, resolved entirely by the end of the treatment period. Initially, the patient struggled with cognitive clarity, impacting his ability to engage in work and daily tasks. By the August 2024 follow-up, cognitive fogginess had significantly diminished, and by November 2024, the patient reported no residual cognitive deficits. The January 2025 assessment confirmed sustained cognitive clarity, with the patient describing enhanced mental sharpness compared to his pre-injury state. This improvement facilitated a return to professional responsibilities and complex decision-making, previously hindered by post-concussive symptoms. The iboga microdosing protocol, combined with AEDP’s focus on emotional processing, likely contributed to this cognitive restoration by enhancing neuroplasticity and reducing psychological barriers to cognitive function.
3. Emotional/Psychological Symptoms: The patient achieved profound emotional and psychological recovery, marked by the complete cessation of anger outbursts that initially occurred multiple times daily. By September 2024, these outbursts had resolved, and the patient developed enhanced emotional awareness and regulation through somatic and mindfulness practices introduced during AEDP therapy. Initial mood lability, irritability, and grief over loss of identity due to lifestyle changes post-TBI gave way to emotional stability and resilience. The patient reported a deepened sense of peace and connection to “something greater,” fostered by ritualized morning routines involving intention setting and gratitude practices. By January 2025, he described his emotional state as “better than ever, and better than before the accident,” reflecting improved interpersonal relationships, particularly with his son, and a renewed enthusiasm for life. The processing of childhood trauma and family dynamics during therapy sessions further supported this emotional transformation, enabling the patient to address long-standing patterns of emotional suppression.
4. Functional Outcomes: The patient’s functional recovery was transformative, enabling a return to meaningful activities and an enhanced quality of life. By August 2024, he resumed physically and emotionally connecting activities, such as hiking with his son, which were previously impossible due to fatigue and irritability. Family dynamics improved significantly, shifting from strained interactions to genuine enjoyment and enthusiasm, as noted by both the patient and his spouse. By November 2024, the patient had self-discontinued all prescription medications, including amitriptyline and Aimovig, relying instead on sustainable lifestyle changes and complementary therapies like sensory deprivation tanks and Lion’s Mane supplementation. Professional functionality was restored, with the patient managing work responsibilities without heat-provoked headache exacerbations. By January 2025, the patient expressed excitement about returning to pre-injury activities and reported a renewed sense of purpose and motivation. The integration of mindfulness practices and improved self-regulation skills supported sustained functional gains, positioning the patient to exceed his pre-injury baseline in overall wellbeing.

## Case report: patient two

### Patient information

A 40 year-old female with a prior medical history of ankylosing spondylitis and depression presented to the neurology clinic with persistent headaches following a hypoxic brain injury sustained during an avalanche in 2016/2017. No direct cranial impact occurred and no helmet damage was observed. The patient was buried beneath snow for approximately 10–15 min, consciousness progressively declined during prolonged hypoxic exposure, respiratory depression was observed upon rescue, and neurocognitive symptoms persisted chronically for approximately 7 years prior to protocol initiation.

### Clinical presentation

The patient initially presented with multiple neurological sequelae following her hypoxic brain injury, including slowness of thought, memory changes, and migraine headaches. While these symptoms showed some improvement over time, she continued to experience significant impairment in her daily functioning.

Two years prior to the most recent evaluation, the patient self-initiated psychedelic therapy with LSD and psilocybin, reporting “massive improvement” in her symptoms. Despite this, she continued to suffer from severe headaches approximately once per month at initial presentation. These headaches were characterized as right-sided, accompanied by photophobia, phonophobia, and nausea, lasting from several hours to an entire day. Her initial self-management strategy involved a combination of acetaminophen, ibuprofen, and diphenhydramine, which typically provided relief after several hours.

The patient also reported persistent “brain fog” with particular difficulty in word finding, which worsened with alcohol consumption. Her sleep was described as difficult, complicated by post-traumatic stress disorder (PTSD) and nightmares, though she noted some improvement with ongoing talk therapy.

### Interval history

During the follow-up visit in August 2024, the patient reported that ubrogepant (Ubrelvy) had been effective when tried as a sample, but insurance approval was pending. Her migraine frequency had increased to five episodes over the previous 2 months, compared to her baseline of once monthly. She had initiated supplements including N-acetylcysteine (NAC), Lion’s Mane mushroom, and magnesium L-threonate, with subsequent improvement in sleep quality. Her cognitive fog remained unchanged. She had successfully completed a slow taper off bupropion (Wellbutrin) without adverse effects.

At her November 2024 follow-up, the patient reported effective abortive treatment of headaches with either ubrogepant (Ubrelvy) or rimegepant (Nurtec), though insurance coverage remained problematic. She disclosed recent unemployment. Her mood was described as stable with anxiety but without depression, and her sleep quality remained variable.

By January 2025, the patient had obtained new insurance coverage and a prescription for ubrogepant had been sent the previous month, though she had not yet filled it. Her headache frequency had decreased to 3–4 episodes per month, with none in the 3 weeks prior to the visit. Notably, she reported resolution of her brain fog.

### Physical examination

In January 2025, vital signs were within normal limits: BP 108/60 mmHg, pulse 68/min, respiratory rate 16/min, and oxygen saturation 95%. The patient was alert and in no acute distress.

### Neurological examination

The patient was alert and in no acute distress. Neurological examination revealed a patient who was alert and oriented to person, place, and time with intact command following. Her speech was normal without dysarthria, and she demonstrated preserved naming and repetition abilities. Cranial nerve assessment showed pupils that were equal, round, and reactive to light bilaterally with full visual fields. Extraocular movements were intact, and she exhibited symmetric facial sensation and movement. Hearing was equal bilaterally to finger rub testing, and her palate elevated symmetrically. Shoulder shrug was full, and her tongue was midline. Motor examination demonstrated 5/5 strength throughout all extremities both proximally and distally with normal tone and no abnormal movements. Sensory examination did not identify any deficits. Deep tendon reflexes were 2+ and symmetric throughout. Coordination testing revealed no ataxia, and the patient ambulated independently without assistance.

### Laboratory and imaging findings

No laboratory results were available for review. Neuroimaging was intentionally deferred, as noted in the assessment and plan.

### Management and treatment course

Initial management included recommendations for sumatriptan (Imitrex) for severe breakthrough headaches, with a plan to transition to rizatriptan (Maxalt) if necessary. A sample of rimegepant (Nurtec) was provided. For cognitive symptoms, N-acetylcysteine and Lion’s Mane supplements were recommended, along with information on sensory deprivation tanks. For insomnia, magnesium L-threonate and/or CBD gummies were suggested, with counseling on sleep hygiene. Continuation of psychological services was recommended for PTSD management.

A treatment addendum in July 2024, noted a recommendation for ubrogepant (Ubrelvy), as the patient experienced four or more migraine days monthly and had failed trials of at least two triptans (sumatriptan and rizatriptan).

By August 2024, improved sleep was reported with magnesium L-threonate supplementation. Ubrogepant remained the recommended abortive treatment for headaches, with additional samples provided. A sample of rimegepant was also provided as an alternative if insurance approval for ubrogepant was denied. Preventive injection therapy was deferred at that time.

The November 2024 assessment noted good response to both ubrogepant and rimegepant for headache management, with continued use of magnesium L-threonate for sleep. The patient was encouraged to implement meditation practices and ensure adequate hydration to address anxiety exacerbated by recent job loss.

At the January 2025 follow-up, samples of ubrogepant were provided while awaiting prescription fulfillment. The clinician documented complete resolution of both brain fog and headaches. Continued hydration and Lion’s Mane supplementation were encouraged.

### Iboga microdosing protocol initiation (October 2024)

Following limited improvement with conventional interventions, the patient was introduced to an iboga microdosing protocol beginning 18 October 2024, with weekly therapeutic sessions performed virtually, scheduled for the subsequent 6 weeks.

### Therapeutic progression

The therapeutic intervention combined iboga microdosing with Accelerated Experiential Dynamic Psychotherapy (AEDP), a somatic-focused approach. Weekly sessions were conducted as follows:

#### Session 1 (October 2024)

Established therapeutic rapport and introduced the iboga protocol, including ritual and spiritual connection aspects. Therapeutic intervention focused on holistic assessment of the patient’s life history, interpersonal relationships, emotional management, and processing of grief related to identity changes following TBI. The patient reported severe PTSD following the TBI with nightmares 1–3 times a month and emotional dysregulation. Experiences chronic fatigue, cognitive fog, and slowness in thought processing. Clarity and alertness are rare, usually only occurring 1–2 days/month. The patient reported strong physical health with an active lifestyle and improved alcohol moderation. Migraines occurred monthly, requiring sensory deprivation, with additional headache days. General emotional instability while navigating complex grief over her mother’s decline and high stress at work. Ttrauma history includes childhood abuse and sexual assault, severe anorexia leading to inpatient treatment at age 20. The behavior has resolved though intrusive thoughts occasionally surface. Has previously received EMDR therapy.

#### Session 2 (October 2024)

Once beginning iboga treatment, the patient experienced headaches resembling a “vice” on her head with mild noise and light sensitivity. Dreams also became more vivid. The patient reported feeling more connected to her body than before. She engaged in grounding exercises to increase bodily awareness. The patient recalled an unexpected memory from childhood accessing sadness and treatment focused on shame, anger, grief, instability, fear, and loss. Significant emotional release followed.

#### Session 3–6 (November 2024)

Treatment used AEDP Psychotherapy to explore traumatic childhood memories across various ages continued in all the following weeks. In particular memories between ages 10–15 that the patient shared had never been revealed including incidents with her alcoholic mother, incarceration of a parent, physical abuse from father, and neglect of basic needs like food and shelter. Iboga created notable access to memories that had not been accessed in decades and processing of emotions like fear, guilt and shame. Significant work done to regulate the overwhelm of emotions, the ability to remain present and connected to the body while experiencing emotion, and narrative re-experiencing of new experiences like safety, trust, co-regulation with another, affirmation, confidence, and care.

Follow Up (January 2025): The patient reported progressive improvement in headaches, emotional regulation, fatigue, and cognitive symptoms following the integrative protocol. The patient reported a significant reduction in frequency of headaches from 3–6 severe headaches monthly to one per month and patient self-reporting iboga greatly improving brain health. Initially iboga led to an increase in headache occurrence then symptoms subsided over time.

In terms of mood, the subject reported a substantial and sustained improvement after iboga, enabling her to discontinue antidepressant medication after 20 years without relapse. She noted enhanced emotional regulation and a new ability to recognize and interrupt depressive spirals. Psychologically, she described reaching a place of acceptance with past trauma and felt more emotionally resilient. Overall, she characterized the change as transformative and described the sensation of “living back in her own brain.”

In regards to the reported history of psychedelic use prior to the iboga microdosing protocol, the patient did not use LSD nor psilocybin during the active iboga-containing treatment interval. Rather, prior intermittent psychedelic exposures occurred years before protocol initiation. Specifically, psilocybin use began in March 2021 and LSD use began in May 2021, whereas the iboga-containing protocol was initiated in October 2024. LSD exposure occurred intermittently, approximately four times annually, outside the active treatment interval and was not co-administered during the protocol.

### Clinical outcome

The patient’s recovery trajectory demonstrated progressive resolution of post-hypoxic injury sequelae symptoms over a 6 month period following the initiation of the iboga microdosing protocol. The integration of conventional neurological care with an iboga microdosing protocol and Accelerated Experiential Dynamic Psychotherapy (AEDP) therapy appeared to address both the neurophysiological and psychological sequelae of chronic hypoxic-ischemic brain injury (HIBI). Notable improvements were observed across multiple domains:

1. Physical Symptoms: The patient experienced a near-complete resolution of headaches, with frequency decreasing from 3–6 severe episodes monthly to one per month by the January 2025 follow-up. Initially, the iboga microdosing protocol led to a temporary increase in headache occurrence, described as a “vice” on her head with mild noise and light sensitivity, but these symptoms subsided over the course of treatment. Chronic fatigue, a significant issue at baseline, was markedly reduced, allowing the patient to resume an active lifestyle. Sleep quality improved with the use of magnesium L-threonate supplementation, and nightmares associated with post-traumatic stress disorder (PTSD) decreased in frequency from 1–3 times monthly to rare occurrences. The patient’s overall physical health remained strong, supported by improved alcohol moderation and enhanced bodily awareness developed through grounding exercises during therapy.
2. Cognitive Symptoms: Cognitive functioning showed substantial improvement, with complete resolution of persistent brain fog, which had been the most debilitating symptom at presentation. The patient reported enhanced memory recall, reading comprehension, and clarity of thought, achieving cognitive alertness on a near-daily basis compared to only 1–2 days per month initially. While the iboga protocol contributed significantly to these improvements, the patient noted a marked cognitive enhancement following self-directed LSD use, which she described as restoring cognitive integration through visual patterning experiences. By the end of the treatment period, the patient reported a return to full cognitive functioning, enabling her to engage in complex tasks without the slowness of thought or word-finding difficulties that characterized her initial presentation.
3. Emotional/Psychological Symptoms: The patient achieved significant emotional stability, culminating in the cessation of antidepressant medication after 20 years of use without relapse. Emotional dysregulation, driven by PTSD and complex grief related to her mother’s decline, improved substantially, with the patient developing the ability to recognize and interrupt depressive spirals. AEDP therapy facilitated the processing of traumatic childhood memories, including abuse, neglect, and parental incarceration, allowing the patient to access and regulate emotions such as fear, guilt, and shame. By the end of treatment, she reported enhanced emotional resilience and a newfound sense of acceptance regarding past trauma. The patient described a transformative psychological shift, characterized by increased confidence, trust, and the ability to co-regulate emotions with others, marking a significant departure from her baseline emotional instability.
4. Functional Outcomes: The patient’s functional recovery was transformative, enabling a return to meaningful activities and improved quality of life. She resumed an active lifestyle, including physical activities previously limited by fatigue and headaches. Social and interpersonal functioning improved as she navigated complex grief and workplace stress with greater emotional regulation. The patient reported enhanced self-care capacity, including the ability to set boundaries and prioritize her needs. Despite recent unemployment, she maintained a stable mood and optimism, reflecting improved coping strategies. The discontinuation of antidepressants and reduced reliance on abortive headache medications (e.g., ubrogepant and rimegepant) underscored her development of sustainable self-management techniques. Overall, the patient described the treatment as a profound return to living “back in her own brain,” reflecting a significant restoration of identity and purpose.

## Case report: patient three

### Patient information and history

A 19 year-old female with no significant past medical history presented to the neurology clinic following consultation for traumatic brain injury (TBI) sustained in a motor vehicle accident (MVA).

### Clinical presentation

The patient was initially evaluated in the emergency department in November 2024, after being involved in an MVA where her vehicle was T-boned at an intersection by another vehicle traveling at approximately 50 mph. She was a restrained driver and denied airbag deployment. At the time of the emergency department presentation, she reported headache located on the superior aspect of her head, mid-back pain, and left-sided chest wall pain. She specifically denied loss of consciousness, nausea, vomiting, dizziness, blurry vision, neck pain, further chest pain, lower back pain, abdominal pain, urinary complaints, or bowel/bladder incontinence.

During her initial neurology clinic visit in December 2024, approximately 2 weeks post-injury, the patient’s clinical presentation had evolved significantly. She reported blurred vision, chronic fatigue, and cognitive difficulties described as “feeling drunk 90% of the time” with confusion. She also noted personality changes including irritability, emotional volatility, and being “less reserved than usual” with “no filter.” The patient described headaches occurring 2–3 times per week, located frontally, throbbing in quality, severe in intensity, and lasting up to 2 days. Acetaminophen provided no relief. She reported nausea and vomiting, alongside both intermittent lightheadedness and vertigo. The patient also described dropping objects and experiencing intermittent numbness in her right hand.

Her sleep patterns were significantly disrupted, requiring 4–6 h naps daily in addition to sleeping 12 h nightly. She acknowledged significant screen time exposure and reported that a 90 min session at a sensory deprivation facility (Healing One) the previous week had provided temporary benefit, described as “clarity of thought for 2 h”

### Interval history

At her follow-up visit on 22 January 2025, approximately 2 months post-injury, the patient reported medication intolerances. Sumatriptan (Imitrex) caused increased lethargy and worsened hand numbness without alleviating her headaches. Rizatriptan (Maxalt), initiated on 19 December 2024, produced extreme fatigue and lethargy with worsening headache symptoms. Ubrogepant (Ubrelvy), prescribed in January 2025, provided no benefit. Her headache frequency had increased to 2–4 episodes weekly, with a continuous headache for the 4 days preceding her visit. She continued to experience excessive sleep requirements and reported poor memory, noting an episode where she “got lost” at school. The patient described anxiety and depression related to her diagnosis. A trial of a sensory deprivation tank had provoked anxiety. She inquired about iboga as a potential treatment, noting her father’s positive experience with this substance for TBI.

By her February 2025 follow-up, approximately 3 months post-injury, the patient reported self-sourcing iboga and following a microdosing protocol similar to her father’s. She was in week 4 of a planned 6 week protocol. At this visit, she reported a continuous headache for the previous 3–4 days, located at the crown and worsening with postural changes such as bending down. The headache was described as pressure-like in quality and moderate in intensity. Rimegepant (Nurtec) had provided no relief, while ubrogepant had offered some improvement.

The patient reported significant symptom improvement across multiple domains. Her fatigue and confusion had substantially improved, now describing herself as 45% symptomatic compared to 90% in December 2024. Emotional lability and volatility had improved by approximately 70%, an assessment her accompanying father agreed with emphatically. Nausea and vomiting had completely resolved. Sleep requirements had decreased to 9–10 h nightly with rare naps (approximately twice weekly, lasting 4–6 h). Lightheadedness, vertigo, and right-hand numbness had all resolved. Headache frequency had decreased substantially, with only one self-resolving episode in the preceding 4 weeks. A second experience with a sensory deprivation tank had been beneficial without provoking anxiety. The patient also reported pursuing talk therapy.

### Physical examination

In February 2025, vital signs showed blood pressure 136/66 mmHg, pulse 90/min, respiratory rate 18/min, and oxygen saturation 98%. The patient’s height was 170.2 cm (5′7.01″) and weight 54.4 kg (120 lb). General examination revealed a patient who was awake and in no acute distress. Cardiovascular examination demonstrated regular rate and rhythm. Neurological examination showed a patient who was awake, alert, and oriented to person, place, and time with intact command following. Her speech was normal without dysarthria, and she demonstrated preserved naming and repetition abilities. Cranial nerve assessment revealed pupils that were equal, round, and reactive to light bilaterally with full visual fields. Extraocular movements were intact with normal convergence and normal saccades. She exhibited symmetric facial sensation and movement. Hearing was equal bilaterally to finger rub testing, and her palate elevated symmetrically. Shoulder shrug was full, and her tongue was midline. Motor examination demonstrated 5/5 strength throughout all extremities both proximally and distally with normal tone and no abnormal movements. Sensory examination did not reveal any deficits. Deep tendon reflexes were 2+ and symmetric throughout. Coordination testing showed no bilateral ataxia on finger chase testing, and the patient ambulated independently without assistance.

### Laboratory and imaging findings

A qualitative urine pregnancy test performed in May 2024 (prior to the accident) was negative.

Computed tomography (CT) of the head performed in December 2024, revealed no acute intracranial abnormality.

Magnetic resonance imaging (MRI) of the brain obtained in January 2025, demonstrated no acute intracranial process, with incidental finding of mild maxillary sinus mucosal thickening.

### Management and treatment course

Initial management included recommendations for an MRI brain (which was subsequently performed), sensory deprivation tank sessions, screen time minimization, alcohol avoidance, N-acetylcysteine (NAC) supplementation, adequate hydration, and conservative management. Sumatriptan was recommended for severe headaches, alongside meditation practice and a diet high in antioxidants.

At the January 2025 follow-up, given the patient’s intolerance to multiple abortive medications and worsening headache frequency and intensity, management was adjusted. The treatment plan included transitioning from ubrogepant to rimegepant for breakthrough headaches, with samples provided. A sample of fremanezumab (Ajovy) was also provided for prevention. A methylprednisolone dose pack was recommended to abort the current cycle of status migrainosus. The clinician provided academic information from medical literature regarding iboga following the patient’s inquiry, while clarifying inability to prescribe this Schedule I substance.

By the February 2025 visit, the patient had self-initiated iboga microdosing with substantial symptomatic improvement. The clinician documented inability to formally recommend this therapeutic but offered to provide academic information from the medical literature. A single 30 mg intramuscular dose of ketorolac (Toradol) was administered during the visit to address status migrainosus.

### Therapeutic progression

The therapeutic intervention combined iboga microdosing with Accelerated Experiential Dynamic Psychotherapy (AEDP). Weekly sessions were conducted as follows:

### Iboga microdosing protocol initiation (February 2025)

#### Session 1 (February 2024)

Established therapeutic rapport and introduced the medicine protocol. Therapeutic intervention focused on holistic assessment of the patient’s life history, interpersonal relationships and emotional management following TBI. The patient reported a significant increase in migraine frequency following the motor vehicle accident. Prior to the accident, she experienced occasional stress-induced headaches but no major neurological concerns. Post-accident, migraines escalated to multiple occurrences per week, described as debilitating and leading to extensive sleep and exhaustion. The patient also noted confusion, disorientation and memory loss.

Inter-relationally, the patient described difficulties with processing social interactions, leading to feelings of awkwardness. The patient also reported increased isolation since the accident and difficulty relating to others. Lifestyle transition from an active, social lifestyle to one with increased restrictions led to a sense of disconnection. Treatment included somatic and parts work which opened into a space of recognizing emotional suppression and culminated in episodes of crying and emotional release linked to frustration and exhaustion. Surfaced acknowledging of anger regarding the disruption to her life and loss of control. Additional therapeutic treatment through AEDP Psychotherapy to create safety with a regulated presence of another to allow and process emotions rather than suppress them.

#### Session 2 (February 2024)

Patient revealed notable shifts in emotional regulation and somatic awareness describing a shift from reactivity to calmness. Reported improvement of energy levels and increased awareness of body, sensations and needs. Cognitive effects were mixed, with reported instances of disorientation and becoming lost in a familiar space. Therapeutic treatment included explorations around fear of unpredictable parental emotions, the death of her grandmother, and the absence of emotional expression. Therapeutic breakthrough with the patient coming to terms with expressing the anger and fear at the impact of the accident on her life, the loss of control, and the fear of not improving. The patient reported a profound sense of peacefulness.

#### Session 3 (February 2024)

The patient reported overwhelming academic and professional responsibilities in the aftermath of the accident. Treatment focused on exploring the somatic sensations of anxiety, nausea and abdominal tension which surfaced the processing of mothers multiple cancer diagnoses and grandmothers passing. The session focused on increasing tolerance for emotional distress and the patient recognized emotional suppression as a longstanding coping mechanism, originating in childhood as a response to overwhelming circumstances. The patient noted a remarkable shift in change of emotional response from distress to curiosity, presence and peace in a distressing situation. Progress in her ability to remain with emotions rather than avoid them, marking an emerging sense of agency in emotional regulation.

#### Session 4 (March 2024)

Notable improvements in migraine symptoms from previous severity levels of self reported 9–10 down to 5–6, enabling functional activities like hiking that were previously impossible. Therapeutic work centered on somatic awareness and brought forward minimized traumatic memory when the patient was drugged without consent (“roofied”), resulting in a 4 h memory gap, 15 h of severe vomiting and hospital submission. Treatment focused on accessing overwhelm, fear, anger, confusion, and aloneness. Through guided imagery and somatic processing released adaptive action tendencies and restored nervous system regulation.

#### Session 5 (March 2024)

Session five documented a notable shift in the patients saying, “I feel like a return to myself pre-accident.” The patient noted new and improved memory functions. Treatment focused on actual processing of the accident and the recognition of significant attachment injury with family members and the long-lasting impact on family relationships. Treatment focused on surfacing deep emotions of anger, resentment, sadness, grief, and aloneness.

#### Session 6 (March 2024)

Significant neuropsychological and interpersonal developments. Cognitive changes included happiness, a sense of euphoria, and enhanced perspective-taking abilities and increased awareness of others’ experiences. Academic functioning showed improvement with faster information absorption. Concurrently, the client experienced mild cognitive disruptions including conversational memory lapses and increased clumsiness. The session revealed significant progress in emotional processing capacity with the patient’s initiating relational repair conversations with parents. Most remarkable was improved affect regulation with anger—with self-regulated processes of acknowledgment, expression, natural de-escalation, and resolution—a process previously inaccessible. Patient expressed optimism regarding future plans, perceiving them as an avenue for personal exploration and growth and gratitude for being a “completely different person.”

### Follow up (June 2025)

Overall, recent months showed high levels of physical energy and participation in life including hiking, swimming, and rock climbing. Recent weeks have seen an increase in frequency of headaches to 2–3 times per week, typically beginning with light pressure and lasting for a day. The past month was also characterized with more emotional dysregulation, mood swings, indecision, and anxiety. The patient is also experiencing feeling sensitive to environmental changes like heat, being drained by schoolwork and inability to retain information. The patient expressed interest in participating in another microdosing protocol with iboga.

### Clinical outcome

The patient’s recovery trajectory demonstrated partial symptomatic improvement over a 3 month period following injury; however, sustained remission was not achieved. Follow-up assessment in June 2025 documented recurrence of headaches, emotional dysregulation, cognitive fatigue, and environmental sensitivity, suggesting incomplete recovery and possible relapse of symptoms consistent with the known natural history of post-concussive syndromes. The patient’s trajectory may partially reflect the expected natural recovery curve observed in mild traumatic brain injury.

Physical symptoms showed remarkable resolution throughout the treatment course. The patient experienced complete resolution of nausea, vomiting, lightheadedness, and vertigo that had significantly impacted her daily functioning at initial presentation. Headache frequency dramatically decreased from 2–4 episodes weekly to just one self-resolving episode over a 4 week period. The right-hand numbness and coordination issues that initially caused her to drop objects completely resolved. Sleep patterns normalized substantially; initially requiring 12 h nightly plus 4–6 h daily naps, the patient’s requirements decreased to 9–10 h nightly with only occasional naps. Energy levels improved considerably with substantially reduced fatigue. Additionally, the patient developed enhanced somatic awareness of body sensations and needs, which appeared to contribute to her improved self-regulation.

1. Cognitive symptoms demonstrated significant improvement from baseline. The patient’s initial presentation of confusion, described as “feeling drunk 90% of the time,” improved to just 45% symptomatic by the end of the treatment period. Memory function improved substantially, with the patient reporting a sense of “return to myself pre-accident.” Information processing and academic functioning enhanced beyond the temporary relief previously experienced with sensory deprivation therapy. The patient demonstrated faster information absorption and improved learning capacity, along with regained ability to navigate familiar environments. Some mild cognitive disruptions persisted, including occasional conversational memory lapses, representing an area for continued therapeutic focus.
2. Emotional and psychological symptoms showed profound improvement. The patient experienced 70% improvement in emotional lability and volatility as confirmed by both self-report and family observation. She developed significantly improved affect regulation, particularly with anger management, transitioning from emotional avoidance to a capacity for effective emotional processing. Anxiety associated with sensory deprivation experiences completely resolved, allowing her to benefit from this complementary therapy. Familial relationships improved markedly, with the patient initiating repair of interpersonal conflicts. She developed emotional regulation skills including acknowledgment, expression, natural de-escalation, and resolution—a process previously inaccessible to her. Irritability decreased alongside improved tolerance for emotional distress. Perhaps most notably, positive emotional states emerged including happiness, optimism, and gratitude, representing a significant contrast to her initial presentation.
3. Functional outcomes demonstrated meaningful clinical improvement. The patient returned to previously impossible physical activities such as hiking. Academic functioning improved with enhanced capacity to manage coursework and information processing. Social functioning normalized with reduced awkwardness in interactions. Self-care capacity and boundary setting improved substantially. The patient showed increased independence in daily activities and renewed ability to engage in future planning with optimism. Family relationships improved through meaningful communication and conflict resolution. Dependence on pharmacological interventions for symptom management decreased as the patient developed sustainable coping strategies for ongoing challenges. Overall, the patient experienced what she described as a significant transformation with substantial quality of life improvement, reflected in her statement of feeling like “a completely different person.” A qualitative summary of symptom trajectories across physical, cognitive, and emotional domains for the three patients is presented in Table 2.

**TABLE 2:** Qualitative symptom trajectory summary.

Domain	Patient 1 (TBI)	Patient 2 (HIBI)	Patient 3 (TBI)
Headache/migraine	Daily → rare (11 months)	3–6/mo → 1/mo	2–4/wk → 1 episode (then relapse)
Brain fog/cognition	Resolved; enhanced sharpness	Complete resolution (LSD + iboga)	90% → 45% symptomatic (partial)
Emotional regulation	Anger resolved; “better than ever”	Off antidepressant 20 years; resilient	70% improvement (relapse at 6 months)
Medications discontinued	Amitriptyline, erenumab-aooe	Antidepressant (20 years)	None sustained

## Discussion

This case series presents a compelling narrative of neurological recovery using a novel iboga microdosing protocol for two patients experiencing prolonged post-concussive syndrome following traumatic brain injury, and one patient with chronic hypoxic-ischemic brain injury (HIBI) refractory to conventional measures. The patients’ progressive improvements over a 9 month period, status post the microdosing protocol, underscores the potential therapeutic value of this innovative approach.

The iboga microdosing intervention, characterized by a carefully structured regimen of sub-perceptual ibogaine doses, demonstrates promising neurological rehabilitation potential (Kuypers, 2020; Winkelman, 2014). The dosing protocol of 4 days on and 3 days off over a 6-week duration minimized potential adverse effects, while potentially leveraging the compound’s neuroplasticity-enhancing properties (Maciulaitis et al., 2008; Ma et al., 2023; Cameron et al., 2021).

### Neurological mechanisms and recovery

The complex pharmacological profile of iboga suggests multiple mechanisms contributing to neurological recovery. Studies have demonstrated its ability to modulate growth factors like GDNF and BDNF in brain regions involved in dopaminergic circuits (He and Ron, 2006), which may be particularly relevant in TBI rehabilitation. The compound’s interaction with neuroplasticity pathways could potentially facilitate neural repair and functional restoration (Ma et al., 2023; Cameron et al., 2021). Future work might include EEG recordings to examine signature ‘psychedelic’ related brain activity changes, such as the ‘entropic brain effect’ (Blest-Hopley et al., 2025).

Traumatic brain injury (TBI) and hypoxic brain injury (HBI) involve distinct yet partially overlapping pathophysiologic mechanisms that may differentially influence neurocognitive outcomes and recovery trajectories. TBI is characterized primarily by biomechanical injury processes including axonal shear stress, focal contusion, microvascular disruption, blood-brain barrier dysfunction, and neuroinflammatory cascades resulting from direct mechanical force. In contrast, hypoxic injury involves diffuse cerebral oxygen deprivation leading to metabolic failure, mitochondrial dysfunction, glutamate-mediated excitotoxicity, oxidative stress, and widespread neuronal vulnerability in regions with high metabolic demand. Hippocampal structures, particularly the CA1 region, demonstrate heightened susceptibility to hypoxic injury and may contribute prominently to persistent impairments in memory consolidation, concentration, and cognitive processing speed. Cortical association networks and watershed regions may likewise exhibit selective vulnerability following prolonged hypoxic exposure. Although the injury mechanisms differ substantially, both TBI and hypoxic injury may converge upon downstream neuroinflammatory and neuroplasticity-related pathways, including dysregulation of brain-derived neurotrophic factor (BDNF) and glial cell line-derived neurotrophic factor (GDNF) signaling. Consequently, while mechanistic extrapolation from TBI and addiction-focused ibogaine literature to chronic hypoxic injury remains highly preliminary, neurotrophic and network-level plasticity pathways may represent biologically plausible areas for future investigation across both injury contexts.

The clinical progression for all three reported patients is notable. Initial presentations of daily headaches, disequilibrium, and fatigue gradually resolved, with the final follow-up revealing substantially and profound reduction in symptomatology. This trajectory aligns with emerging research exploring psychedelic interventions for neurological recovery, particularly in veteran populations (Brody and Siddiqi, 2024; Cherian K. N. et al., 2024; Khan et al., 2022).

### Safety considerations

While the case series highlights promising outcomes, it's crucial to acknowledge the potential risks associated with ibogaine. Previous research has documented concerns about cardiac effects, including QT interval prolongation and potential arrhythmias (Koenig and Hilber, 2015; Litjens and Brunt, 2016; Knuijver et al., 2024; Kubiliene et al., 2008; Knuijver et al., 2022; Mash et al., 2000). The careful screening procedure employed in this study, which excluded patients with cardiac conditions and other contraindications, represents a critical risk mitigation strategy (Winkelman, 2014; Mash et al., 1998). The microdosing protocol was implemented acknowledging medical background screening for potential drug interactions, and past medical history contraindications including heart conditions and/or the concomitant administration of selective serotonin reuptake inhibitors (SSRIs) (Cherian K. et al., 2024).

The involvement of indigenous healers for ongoing monitoring and support adds an important layer of holistic care and potentially contributes to the intervention’s efficacy. This approach resonates with emerging ethical principles in psychedelic research that emphasize traditional knowledge and comprehensive patient support (Loizaga-Velder and Loizaga Pazzi, 2024).

### Comparison to emerging psychedelic therapies

Complementing the potential neuroplastic and anti-inflammatory effects observed in our iboga microdosing cases, recent reviews highlight the promise of other serotonergic psychedelics, such as psilocybin and 5-methoxy-N,N-dimethyltryptamine (5-MeO-DMT), in alleviating TBI-related impairments through enhanced neuroplasticity, reduced neuroinflammation, and neurotrophic actions (e.g., via TrkB receptor targeting by psilocybin and sigma-1 receptor modulation by 5-MeO-DMT), underscoring the broader therapeutic potential of this class for post-concussive recovery (Blest-Hopley et al., 2025).

Recent observational evidence from veterans with a history of traumatic brain injury participating in psilocybin retreats further supports the potential of serotonergic psychedelics for this population, demonstrating substantial reductions in PTSD (50% decrease in PCL-5 scores), depression (65% decrease in PHQ-9 scores), and anxiety symptoms, alongside normalized spontaneous EEG activity (e.g., reduced delta and theta power in frontal/temporal regions indicating improved emotional processing and cognitive control, with enhanced alpha/beta coherence suggesting better neural connectivity)—outcomes that parallel the symptomatic and functional improvements reported in our iboga microdosing cases and highlight shared therapeutic pathways in prolonged post-concussive recovery (Lissemore et al., 2025).

Building on these serotonergic parallels, recent neurophysiological evidence from veterans with traumatic brain injury treated with magnesium-ibogaine demonstrates a single-session ‘slowing’ of cortical oscillations—characterized by increased power in slower theta-alpha bands, decreased beta-gamma power, an elevated theta/beta ratio correlating with improved cognitive inhibition, and persistently reduced neural complexity (lower spatiotemporal signal entropy)—which associates with sustained enhancements in executive function, PTSD, and anxiety symptoms; these objective EEG changes provide mechanistic insight into ibogaine’s potential to reorganize disrupted brain networks in TBI, offering convergent support for the symptomatic improvements observed in our iboga microdosing protocol and highlighting avenues for future biomarker integration in prolonged post-concussive research (Marton et al., 2019).

### Comparative context and future directions

While this is a single case series of the patients demonstrating profound benefit, the work contributes to a growing body of research exploring alternative therapeutic approaches for TBI (Brody and Siddiqi, 2024; Cherian K. N. et al., 2024; Khan et al., 2022). The multimodal approach, which included supplementation and alternative therapies alongside iboga microdosing, suggests the potential value of integrated rehabilitation strategies.

### Future research should focus on

1. Expanding cohort sizes to validate these preliminary findings.
2. Developing standardized protocols for iboga microdosing in neurological rehabilitation.
3. Comprehensive long-term follow-up to assess sustained neurological improvements.
4. Detailed neuroimaging and neuropsychological assessments to quantify recovery mechanisms.

### Limitations and future directions

The primary limitation of this case series is its small sample size, lack of randomization or blinding, and anecdotal nature. While offering valuable insights, broader clinical validation is necessary. Additionally, the potential placebo effect and the patient’s concurrent therapeutic interventions cannot be definitively isolated.

The observed neurological and functional improvements in these cases with prolonged post-concussive syndrome raise the possibility that iboga microdosing may exert therapeutic effects through promotion of neuroplasticity and neural repair. Preclinical studies in rodents have demonstrated that ibogaine administration upregulates glial cell line-derived neurotrophic factor (GDNF) selectively in dopaminergic regions such as the ventral tegmental area (VTA) and substantia nigra, as well as brain-derived neurotrophic factor (BDNF) across mesocorticolimbic circuits including the nucleus accumbens, prefrontal cortex, and substantia nigra (Palmer et al., 2025). These neurotrophic changes support dopaminergic neuron survival, enhance synaptic plasticity, and may facilitate restoration of disrupted reward and motivational pathways—mechanisms potentially relevant to the persistent cognitive, emotional, and somatic symptoms following traumatic brain injury.

Future research should investigate whether iboga microdosing modulates GDNF and BDNF levels in humans with traumatic brain injury and prolonged post-concussive syndrome, and whether such changes correlate with neural repair (e.g., via neuroimaging markers of white matter integrity or functional connectivity) and functional restoration. Controlled trials incorporating biomarkers of neurotrophic activity, advanced neuroimaging (e.g., diffusion tensor imaging, resting-state fMRI), and longitudinal assessments could elucidate these mechanisms and build on the promising multi-modal, holistic approach described here, including integration of ceremonial frameworks, parts work, and Accelerated Experiential Dynamic Psychotherapy (AEDP).

As with all case series, particularly in the emerging domain of psychedelic interventions for traumatic brain injury and prolonged post-concussive symptoms, several interpretive boundaries must be acknowledged to contextualize these preliminary observations. First, natural recovery trajectories in persistent post-concussive symptoms can extend over months to years, with a subset of individuals experiencing gradual improvement even without novel interventions; consensus guidelines indicate that while most concussion symptoms resolve within weeks to 3 months, 10%–30% develop persisting symptoms beyond this period, and spontaneous resolution or adaptation remains possible in prolonged cases. The improvements observed here could thus partly reflect ongoing natural recovery processes, though the chronicity and severity of symptoms prior to initiation of the iboga microdosing protocol (persisting for months to years despite prior care) suggest contributions beyond typical resolution.

Second, participants received concurrent multidisciplinary support, including weekly counseling, therapeutic alliance-building, parts work, Accelerated Experiential Dynamic Psychotherapy (AEDP), and integration of ceremonial/ritual elements from Sangoma healers. These holistic components, while integral to the protocol, introduce challenges in isolating the specific causal role of iboga microdosing from nonspecific therapeutic factors or the synergistic effects of combined modalities.

Third, assessments relied primarily on clinical observations, standardized patient-reported symptom scales (e.g., Rivermead Post-Concussion Symptoms Questionnaire), and functional outcomes, which are inherently subjective and susceptible to reporting biases. Objective biomarkers (e.g., advanced neuroimaging or neurotrophic factor levels) were not systematically incorporated, limiting mechanistic inference.

Finally, as in much early psychedelic research, selection bias (e.g., motivated individuals seeking novel treatments) and expectancy effects may have influenced outcomes. Positive expectations surrounding psychedelics, amplified by media attention and the therapeutic reputation of iboga, can contribute to perceived benefits independent of or synergistic with pharmacological actions, a phenomenon well-documented in psychedelic trials where unblinding and expectancy often enhance apparent efficacy.

These considerations represent shared constraints in hypothesis-generating case series and observational studies in this nascent field, rather than unique shortcomings of the present work. They underscore the preliminary nature of the findings and highlight the critical need for controlled, prospective trials to disentangle specific drug effects from contextual and recovery-related factors.

## Conclusion

As research in this field continues to expand, it becomes increasingly important for primary care clinicians to understand the therapeutic potential and clinical considerations of ibogaine-based interventions (Lissemore et al., 2025). The observed neurological recovery suggests iboga microdosing warrants further systematic investigation as a potential therapeutic approach for TBI rehabilitation. The patient’s progressive symptom resolution, coupled with the intervention’s apparent safety when carefully implemented, presents an intriguing avenue for future neurological research (Luz and Mash, 2021; Mash et al., 2018). The promising outcomes of iboga microdosing in this case series, alongside emerging research on psilocybin’s potential in TBI recovery, underscore the need for further exploration of psychedelic compounds as novel therapeutic options (Palmer et al., 2025). Palmer et al. (2025) suggest that psychedelics inclusive of psilocybin may enhance neural repair, providing a comparative context for iboga’s observed effects and warrants rigorous clinical trials (Palmer et al., 2025).

Recent observational findings from the Global Psychedelic Survey suggested that individuals with traumatic brain injury histories reported perceived improvements across cognitive, emotional, and somatic symptom domains following psychedelic use, further reflecting growing interest in exploratory psychedelic-assisted neurorehabilitation paradigms (VanderZwaag et al., 2026). Controlled prospective investigations incorporating standardized neuropsychiatric outcomes, objective biomarkers, and longitudinal safety monitoring will be necessary to clarify the therapeutic potential, mechanistic basis, and safety profile of iboga-containing interventions in persistent post-concussive and hypoxic brain injury syndromes.

### Commentary on iboga root bark biomass vs. ibogaine isolate

Unlike purified ibogaine hydrochloride, whole Tabernanthe iboga root bark biomass contains a complex mixture of naturally occurring indole alkaloids, including ibogaine, noribogaine, tabernanthine, ibogaline, voacangine, and related compounds that may collectively contribute to the pharmacologic and experiential profile of iboga preparations (Popik and Skolnick, 1999; Antonio et al., 2013). While ibogaine remains the most extensively studied constituent, emerging ethnopharmacologic and pharmacologic literature has increasingly emphasized that whole-plant iboga preparations may not be mechanistically equivalent to isolated ibogaine exposure. The presence of multiple alkaloids with partially overlapping serotonergic, glutamatergic, opioid, sigma-receptor, and neurotrophic signaling properties raises the possibility of an “entourage effect,” whereby synergistic or modulatory interactions among alkaloid constituents may influence tolerability, duration of effect, subjective phenomenology, and potential neuroplastic outcomes (Mosca et al., 2023). Noribogaine, in particular, demonstrates distinct pharmacokinetic and receptor-binding characteristics relative to ibogaine, including prolonged serotonergic and opioid-modulatory activity that may contribute to sustained neurobehavioral effects (Baumann et al., 2000). Consequently, mechanistic extrapolation from purified ibogaine literature to whole root bark biomass preparations should be interpreted cautiously, and future studies directly comparing isolated ibogaine to whole-alkaloid iboga preparations may be important for clarifying differential therapeutic and safety profiles.

## Data Availability

The original contributions presented in the study are included in the article/supplementary material, further inquiries can be directed to the corresponding author.

## References

[B1] Antonio T. Childers S. R. Rothman R. B. Dersch C. M. King C. Kuehne M. (2013). Effect of iboga alkaloids on µ-opioid receptor-coupled G protein activation. PLoS One 8 (10), e77262. 10.1371/journal.pone.0077262 24204784 PMC3818563

[B2] Baumann M. H. Pablo J. P. Ali S. F. Rothman R. B. Mash D. C. (2000). Noribogaine (12-hydroxyibogamine): a biologically active metabolite of the antiaddictive drug ibogaine. Ann. N. Y. Acad. Sci. 914, 354–368. 10.1111/j.1749-6632.2000.tb05210.x 11085335

[B3] Blest-Hopley G. Pasculli G. Ruffell S. G. D. Tsang W. Emmanuel O. Pate K. M. (2025). Improved mental health outcomes and normalised spontaneous EEG activity in veterans reporting a history of traumatic brain injuries following participation in a psilocybin retreat. Front. Psychiatry 16, 1594307. 10.3389/fpsyt.2025.1594307 40842948 PMC12364870

[B4] Brody D. L. Siddiqi S. H. (2024). An ancient psychedelic for traumatic brain injury. Nat. Med. 30 (2), 342–343. 10.1038/s41591-023-02759-w 38287167

[B5] Brown T. K. Alper K. (2018). Treatment of opioid use disorder with ibogaine: detoxification and drug use outcomes. Am. J. Drug Alcohol Abuse 44 (1), 24–36. 10.1080/00952990.2017.1320802 28541119

[B6] Cameron L. P. Tombari R. J. Lu J. Pell A. J. Hurley Z. Q. Ehinger Y. (2021). A non-hallucinogenic psychedelic analogue with therapeutic potential. Nature 589 (7842), 474–479. 10.1038/s41586-020-3008-z 33299186 PMC7874389

[B7] Carhart-Harris R. L. (2018). The entropic brain - revisited. Neuropharmacology 142, 167–178. 10.1016/j.neuropharm.2018.03.010 29548884

[B8] Carhart-Harris R. L. Friston K. J. (2019). REBUS and the anarchic brain: toward a unified model of the brain action of psychedelics. Pharmacol. Rev. 71 (3), 316–344. 10.1124/pr.118.017160 31221820 PMC6588209

[B37] Cherian K. Shinozuka K. Tabaac B. J. Arenas A. Beutler B. D. Evans V. D. (2024). Psychedelic therapy: a primer for primary care clinicians-ibogaine. Am. J. Ther. 31 (2), e133–e140. 10.1097/MJT.0000000000001723 38518270

[B9] Cherian K. N. Keynan J. N. Anker L. Faerman A. Brown R. E. Shamma A. (2024). Magnesium-ibogaine therapy in veterans with traumatic brain injuries. Nat. Med. 30 (2), 373–381. 10.1038/s41591-023-02705-w 38182784 PMC10878970

[B10] de Vos C. M. H. Mason N. L. Kuypers K. P. C. (2021). Psychedelics and neuroplasticity: a systematic review unraveling the biological underpinnings of psychedelics. Front. Psychiatry 12, 724606. 10.3389/fpsyt.2021.724606 34566723 PMC8461007

[B11] He D. Y. Ron D. (2006). Autoregulation of glial cell line-derived neurotrophic factor expression: implications for the long-lasting actions of the anti-addiction drug, ibogaine. FASEB J. 20 (13), 2420–2422. 10.1096/fj.06-6394fje 17023388

[B12] Khan A. J. Bradley E. O'Donovan A. Woolley J. (2022). Psilocybin for trauma-related disorders. Curr. Top. Behav. Neurosci. 56, 319–332. 10.1007/7854_2022_366 35711024

[B13] Knuijver T. Schellekens A. Belgers M. Donders R. van Oosteren T. Kramers K. (2022). Safety of ibogaine administration in detoxification of opioid-dependent individuals: a descriptive open-label observational study. Addiction 117 (1), 118–128. 10.1111/add.15448 33620733 PMC9292417

[B14] Knuijver T. Ter Heine R. Schellekens A. F. A. Heydari P. Lucas L. Westra S. (2024). The pharmacokinetics and pharmacodynamics of ibogaine in opioid use disorder patients. J. Psychopharmacol. 38 (5), 481–488. 10.1177/02698811241237873 38519421 PMC11102648

[B15] Koenig X. Hilber K. (2015). The anti-addiction drug ibogaine and the heart: a delicate relation. Molecules 20 (2), 2208–2228. 10.3390/molecules20022208 25642835 PMC4382526

[B16] Kubiliene A. Marksiene R. Kazlauskas S. Sadauskiene I. Razukas A. Ivanov L. (2008). Acute toxicity of ibogaine and noribogaine. Med. Kaunas. 44 (12), 984–988. 10.3390/medicina44120123 19142057

[B17] Kuypers K. P. C. (2020). The therapeutic potential of microdosing psychedelics in depression. Ther. Adv. Psychopharmacol. 10, 2045125320950567. 10.1177/2045125320950567 32922736 PMC7457631

[B18] Lissemore J. I. Chaiken A. Cherian K. N. Buchanan D. Espil F. Keynan J. N. (2025). Magnesium–ibogaine therapy effects on cortical oscillations and neural complexity in veterans with traumatic brain injury. Nat. Ment. Health 3 (8), 918–931. 10.1038/s44220-025-00463-x

[B19] Litjens R. P. Brunt T. M. (2016). How toxic is ibogaine? Clin. Toxicol. (Phila) 54 (4), 297–302. 10.3109/15563650.2016.1138226 26807959

[B20] Loizaga-Velder A. Loizaga Pazzi A. (2024). Traditional medicine, culture, and psychedelic science: new pathways for recovery from substance use disorders. J. Stud. Alcohol Drugs 85 (5), 595–606. 10.15288/jsad.23-00011 39400118

[B21] Luz M. Mash D. C. (2021). Evaluating the toxicity and therapeutic potential of ibogaine in the treatment of chronic opioid abuse. Expert Opin. Drug Metab. Toxicol. 17 (9), 1019–1022. 10.1080/17425255.2021.1944099 34139922

[B22] Mastinu A. Anyanwu M. Carone M. Abate G. Bonini S. A. Peron G. (2023). The bright side of psychedelics: latest advances and challenges in neuropharmacology. Int. J. Mol. Sci. 24 (2), 1329. 10.3390/ijms24021329 36674849 PMC9865175

[B23] Maciulaitis R. Kontrimaviciute V. Bressolle F. M. Briedis V. (2008). Ibogaine, an anti-addictive drug: pharmacology and time to go further in development. A narrative review. Hum. Exp. Toxicol. 27 (3), 181–194. 10.1177/0960327107087802 18650249

[B24] Marton S. González B. Rodríguez-Bottero S. Miquel E. Martínez-Palma L. Pazos M. (2019). Ibogaine administration modifies GDNF and BDNF expression in brain regions involved in mesocorticolimbic and nigral dopaminergic circuits. Front. Pharmacol. 10, 193. 10.3389/fphar.2019.00193 30890941 PMC6411846

[B25] Mash D. C. Kovera C. A. Buck B. E. Norenberg M. D. Shapshak P. Hearn W. L. (1998). Medication development of ibogaine as a pharmacotherapy for drug dependence. Ann. N. Y. Acad. Sci. 844, 274–292. 10.1111/j.1749-6632.1998.tb08242.x 9668685

[B26] Mash D. C. Kovera C. A. Pablo J. Tyndale R. F. Ervin F. D. Williams I. C. (2000). Ibogaine: complex pharmacokinetics, concerns for safety, and preliminary efficacy measures. Ann. N. Y. Acad. Sci. 914, 394–401. 10.1111/j.1749-6632.2000.tb05213.x 11085338

[B27] Mash D. C. Duque L. Page B. Allen-Ferdinand K. (2018). Ibogaine detoxification transitions opioid and cocaine abusers between dependence and abstinence: clinical observations and treatment outcomes. Front. Pharmacol. 9, 529. 10.3389/fphar.2018.00529 29922156 PMC5996271

[B28] Mosca A. Chiappini S. Miuli A. Mancusi G. Santovito M. C. Di Carlo F. (2023). Ibogaine/noribogaine in the treatment of substance use disorders: a systematic review of the current literature. Curr. Neuropharmacol. 21 (11), 2178–2194. 10.2174/1570159X21666221017085612 36263479 PMC10556383

[B29] Noller G. E. Frampton C. M. Yazar-Klosinski B. (2018). Ibogaine treatment outcomes for opioid dependence from a twelve-month follow-up observational study. Am. J. Drug Alcohol Abuse 44 (1), 37–46. 10.1080/00952990.2017.1310218 28402682

[B30] O'Hearn E. Molliver M. E. (1993). Degeneration of purkinje cells in parasagittal zones of the cerebellar vermis after treatment with ibogaine or harmaline. Neuroscience 55 (2), 303–310. 10.1016/0306-4522(93)90500-f 8377927

[B31] Palmer C. Ferber A. T. Greenwald B. D. (2025). The potential role of psilocybin in traumatic brain injury recovery: a narrative review. Brain Sci. 15 (6), 572. 10.3390/brainsci15060572 40563744 PMC12190232

[B32] Plummer Z. Allen J. Brand J. Mayo L. M. Shultz S. R. Christie B. R. (2025). Examining the potential of psilocybin and 5-MeO-DMT as therapeutics for traumatic brain injury. Prog. Neuro-Psychopharmacology Biol. Psychiatry 141, 111448. 10.1016/j.pnpbp.2025.111448 40669813

[B33] Popik P. Skolnick P. (1999). Chapter 3 - pharmacology of ibogaine and ibogaine-related alkaloids. Alkaloids Chem. Biol. 52, 197–231.

[B34] VanderZwaag B. Robinson J. Garcia-Romeu A. Garcia-Barrera M. Lake S. Lucas P. (2026). Psychedelics for the management of symptoms of traumatic brain injury: findings from the global psychedelic survey. Prog. Neuropsychopharmacol. Biol. Psychiatry 145, 111624. 10.1016/j.pnpbp.2026.111624 41621525

[B35] Vocci F. J. London E. D. (1997). Assessment of neurotoxicity from potential medications for drug abuse: ibogaine testing and brain imaging. Ann. N. Y. Acad. Sci.; 820:29–39. 10.1111/j.1749-6632.1997.tb46187.x 9237447

[B36] Winkelman M. (2014). Psychedelics as medicines for substance abuse rehabilitation: evaluating treatments with LSD, peyote, ibogaine and ayahuasca. Curr. Drug Abuse Rev. 7 (2), 101–116. 10.2174/1874473708666150107120011.PMID:25563446 25563446

